# TMEM74 promotes tumor cell survival by inducing autophagy via interactions with ATG16L1 and ATG9A

**DOI:** 10.1038/cddis.2017.370

**Published:** 2017-08-31

**Authors:** Yizhe Sun, Yingyu Chen, Jingyu Zhang, Lulu Cao, Minwei He, Xi Liu, Ning Zhao, Ang Yin, He Huang, Lu Wang

**Affiliations:** 1 Peking University Center for Human Disease Genomics, Department of Immunology, Key Laboratory of Medical Immunology, Ministry of Health, School of Basic Medical Sciences, Peking University Health Science Center, Beijing 100191, China; 2 Department of Hematology, Peking University Third hospital, Beijing 100191, China

## Abstract

Autophagy is a highly inducible system of intracellular degradation that occurs in lysosomes or vacuoles. Transmembrane 74 (TMEM74) has been shown to induce autophagy. However, the mechanism by which TMEM74 stimulates autophagy and the impacts of TMEM74-induced autophagy on tumor cell survival remain unclear. In this study, TMEM74 was shown to increase the autophagic flux process in different tumor cell lines. Further investigations revealed that TMEM74 interacts with ATG16L1 and ATG9A. Moreover, distinctive from the common autophagy models, it is found that TMEM74-related autophagy is independent of BECN1/PI3KC3 complex and ULK1, and TMEM74 may initiate and promote autophagy directly via interactions with ATG16L1 and ATG9A responsible for the nucleation and elongation respectively. Considering the ultimate outcome of TMEM74-induced autophagy in tumor cells, TMEM74-triggered autophagy induces a pro-survival effect on tumor cells, particularly cells under metabolic stress, consistent with alteration of a series of signal pathways. Intriguingly, TMEM74 itself can be downregulated through the autophagic process, which indicates that a potential self-regulatory loop exists so as to maintain an appropriate level of autophagy, avoiding excessive autophagy to commit tumor cells to death. According to the clinical database analysis, the high expression of TMEM74 significantly shortens the surviving periods of patients in several specific cancers indicating that TMEM74 itself can be treated as an effective potential target with clinical values to prolong surviving periods of cancer patients in the future. In conclusion, our study reveals a new mechanism by which autophagy is stimulated by a novel positive modulator through a unique pathway and demonstrates a novel connection between autophagy and cell survival, which undoubtedly serves to broaden our understanding of autophagy.

Autophagy (commonly referred to as macroautophagy) is the conserved process by which cells degrade cellular components sequestered by a membranous structure, which is transported to lysosomes for digestion.^1^ Autophagy, which is implicated in a range of pathophysiological conditions, generates metabolic substrates to accommodate cellular bioenergetic demands and maintain intracellular homeostasis.^2, 3^ Additionally, autophagy destroys invasive pathogens, and transfers them to MHC class II compartments to active the immune system.^4, 5, 6^ Thus, basal levels of autophagy exert pro-survival effects on cells. However, autophagy beyond a potential threshold or the induction of uncontrolled autophagy contributes to cell death, which is characterized by the massive accumulation of autophagic structures.^7^ In this circumstance, autophagy is regarded as a self-destruction process, where the cells undergo autophagic cell death, also known as type II programmed cell death.^8, 9, 10^

About thirty autophagy-related proteins (Atgs) have been shown to participate in autophagic process. Most Atg proteins are involved in the phases of autophagosome formation.^11^ The Atg1 complex, which consists of the serine/threonine protein kinase Atg1, has a fundamental role in recruiting numerous proteins to promote autophagy. Upstream activation of the Atg1 complex requires the inhibition of mTOR in response to an environmental change, such as starvation. Under normal conditions, mTOR is a negative modulator of autophagy, as it maintains the hyperphosphorylation of Atg13 to reduce its affinity for Atg1, and thus autophagy is constrained at a baseline level.^12, 13, 14^

The PI3KC3 (Class III PI3K) complex plays an essential role in the process of phagophore nucleation. Additionally, PI3KC3 promotes the recruitment of proteins containing FYVE/PX motifs to increase the size of the sequestered membranes by phosphorylating phosphatidylinositol (PI) to yield phosphatidylinositol-3-phosphate (PI3-P).^15, 16^ Two ubiquitination-like conjugation systems, the Atg5-Atg12/Atg16 complex and the Atg8-phosphatidylethanolamine(PE) complex, mediate the elongation of autophagosomal membranes by a series of E1-like or E2-like enzymes.^17, 18^

Atg9, a transmembrane protein, is required in autophagosomal expansion and transportation of the membranous components by shuttling between autophagosomal intermediates, including PAS (phagophore assembly sites) in yeast and the peripheral compartments.^19, 20^

Transmembrane 74 (TMEM74), which contains two TM domains, was first identified in our lab. TMEM74 can induce autophagy, and is required for EBSS-induced autophagy. However, the mechanism by which TMEM74 regulates cell autophagy and the cell survival outcomes related to TMEM74-triggered autophagy have not yet been investigated.^21, 22^ The present study shows that TMEM74 induces autophagy by interacting with ATG16L1 and ATG9A. The relationship between TMEM74-induced autophagy and tumor cell survival has also been explored, and we generate a novel model concatenating autophagy and tumor cell fate.

## Results

### TMEM74 induces autophagy and increases autophagic flux in different tumor cell lines

**Figure 1:**
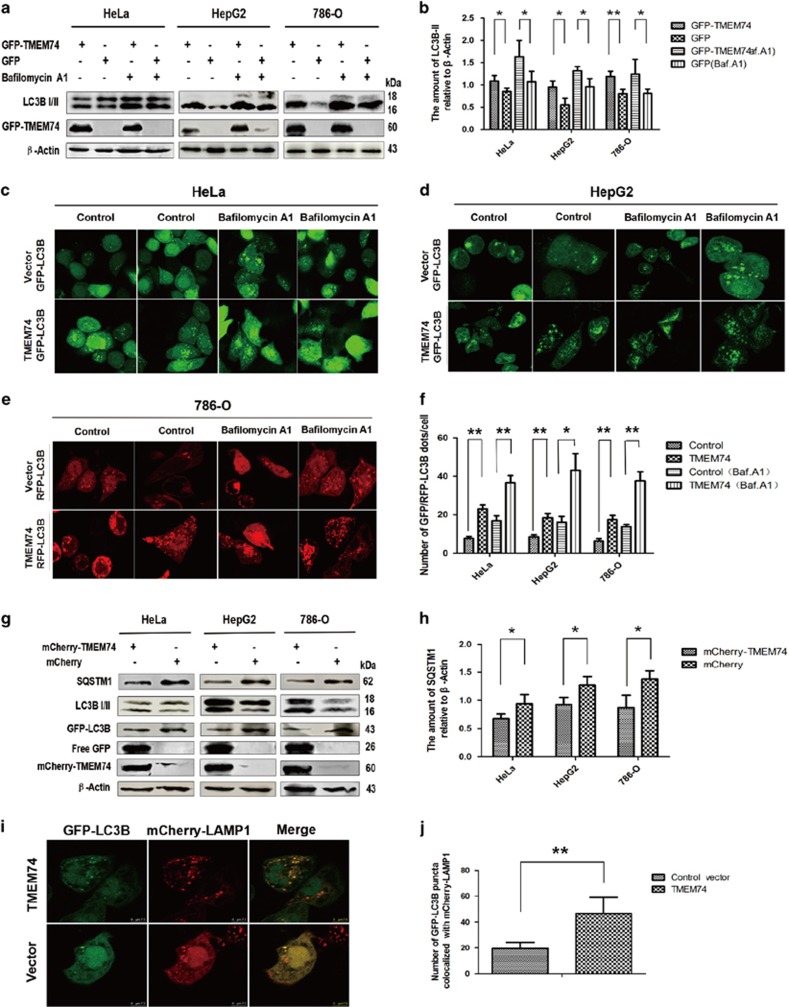
TMEM74 overexpression induces cell autophagy and increases autophagic flux. (**a**) HeLa cells, HepG2 cells and 786-O cells were transfected with GFP-TMEM74 or GFP plasmids (control) for 24 h, and treated with bafilomycin A1 (100 nM) for at least 12 h. The levels of LC3B-II were measured by western blotting. (**b**) Quantification of LC3B-II levels relative to *β*-Actin was shown as column. Data are means±S.D. of three experiments. *P<0.05, **P<0.01. (**c**,**d**) HeLa cells and HepG2 cells were co-transfected with GFP-LC3B and mCherry-TMEM74 or GFP-LC3B and mCherry (control), and treated with Bafilomycin A1 (100 nM) for at least 12 h.Representative confocal microscopy images of GFP-LC3B distribution were shown. (**e**) 786-O cells were co-transfected with GFP-LC3B and GFP-TMEM74 or RFP-LC3B and mCherry (control), and treated with Bafilomycin A1 (100 nM) for at least 12 h.Representative confocal microscopy images of RFP-LC3B distribution were shown. (**f**) Quantification of GFP-LC3B or RFP-LC3B punta per cell was shown as column. Data are means±S.D. of at least 20 cells scored from at least three observed regions.*P<0.05, **P<0.01. (**g**) HeLa cells, HepG2 cells, and 786-O cells were co-transfected with mCherry-TMEM74 and GFP-LC3B or mCherry (control) and GFP-LC3B for 24 h respectively. Levels of SOSTM1, free GFP, and endogenous LC3B-II were analyzed by western blotting. (**h**) Quantification of SQSTM1 levels relative to *β*-Actin was shown as column. Data are means±S.D. of three experiments. *P<0.05, **P<0.01. (**i**,**j**) Colocalization of GFP-LC3B with mCherry-LAMP1 obtained from the HeLa cells co-transfected with TMEM74, GFP-LC3B and mCherry-LAMP1 or control vector, GFP-LC3B and mCherry-LAMP1 for 24 h, respectively, and then observed by confocal microscopy. Quantification of the GFP-LC3B puncta colocalized with mCherry-LAMP1 was shown as column. Data are means±S.D. of the cells from at least three observed regions.*P<0.05, **P<0.01

We conducted the experiments with or without bafilomycin A1 treatment to elucidate the pathway responsible for the TMEM74-induced LC3B increase. In addition to HeLa cells, the experiments were performed in HepG2 cells and 786-O cells. TMEM74 overexpression increased the LC3B-II levels in the three cell lines, and the bafilomycin.A1 treatment resulted in a further increase in the endogenous LC3B-II levels (Figures 1a and b). Similar results were obtained in analyses of GFP/RFP-LC3B puncta distribution (Figures 1c–f).

Next, we examined whether TMEM74 influences autophagic flux. According to the confocal microscopy, the number of GFP-LC3B puncta colocalized with mCherry-LAMP1 increased in TMEM74-transfected HeLa cells compared with the control cells (Figures 1i and j). Simultaneously, TMEM74 overexpression contributed to the increase in free GFP levels and the upregulation of SQSTM1 degradation in the three tumor cell lines (Figures 1g and h). Thus, TMEM74 promotes autophagic flux and autophagolysosome formation.

*TMEM74* knockdown has been shown to hamper EBSS-induced autophagy in HeLa cells. The same experiments were performed and replicated in the other two tumor cell lines (Supplementary Figures 1SA and B).

TMEM74 contains two putative TM domains (amino acid residues 176–198 and 232–252).^22^ To explore whether the TM domains of TMEM74 influenced its function, a plasmid expressing truncated TMEM74 was constructed: TMEM74^△^ (Supplementary Figure 1SC). The autophagic activity of the TMEM74 mutant was tested, and the TMEM74 mutant failed to exert the pro-autophagy effect, as the LC3B-II levels decreased, and sparse LC3 puncta distribution was observed by confocal microscopy (Supplementary Figures 1SD–F). Moreover, GFP-TMEM74^△^ did not display punctate localization, as observed with immunofluorescence microscopy (data not shown). Based on these results, the TM domains are required for TMEM74 function.

### Knockdown of *TMEM74* hampers the LC3 puncta formation and reduces the colocalization between LC3 and ATG16L1 or STX17

Since it is determined that TMEM74 induces autophagy, we next explored the mechanism underlying TMEM74-triggered autophagy. Several autophagic key molecules were chosen to conduct our experiments. ATG16L1 participates at autophagosomal membranes elongation. LC3B spans all phases of autophagosomes and autophgolysosomes formation. STX17, which is recruited to autophagosomal membranes after maturation of autophagosomes and presumed responsible for the incorporation between autophagosomes and lysosomes by interacting with other molecules.^23, 24, 25^ Using confocal microscopy, we observed that knockdown of *TMEM74* reduced the number of RFP-LC3 colocalization puncta with GFP-ATG16L1 and GFP-STX17 significantly; simultaneously, the RFP-LC3 puncta declined under EBSS treatment (Supplementary Figures 2SA–G), which indicates that TMEM74 plays an indispensable role in EBSS-induced autophagy.

We further investigated the localization of TMEM74 besides lysosomes. Based on live images of HeLa cells expressing GFP-TMEM74 and mCherry-ER or mCherry-Mito (two mCherry fluorescent protein-labeled signal peptides), TMEM74 is located in the ER and incrementally associates with the mitochondria in a time-dependent manner (Supplementary Figures 3SA–C), implying that TMEM74 translocation during the autophagic process may exist in the course of autophagy.

### TMEM74 interacts with ATG16L1 via the WD domain of ATG16L1

**Figure 2:**
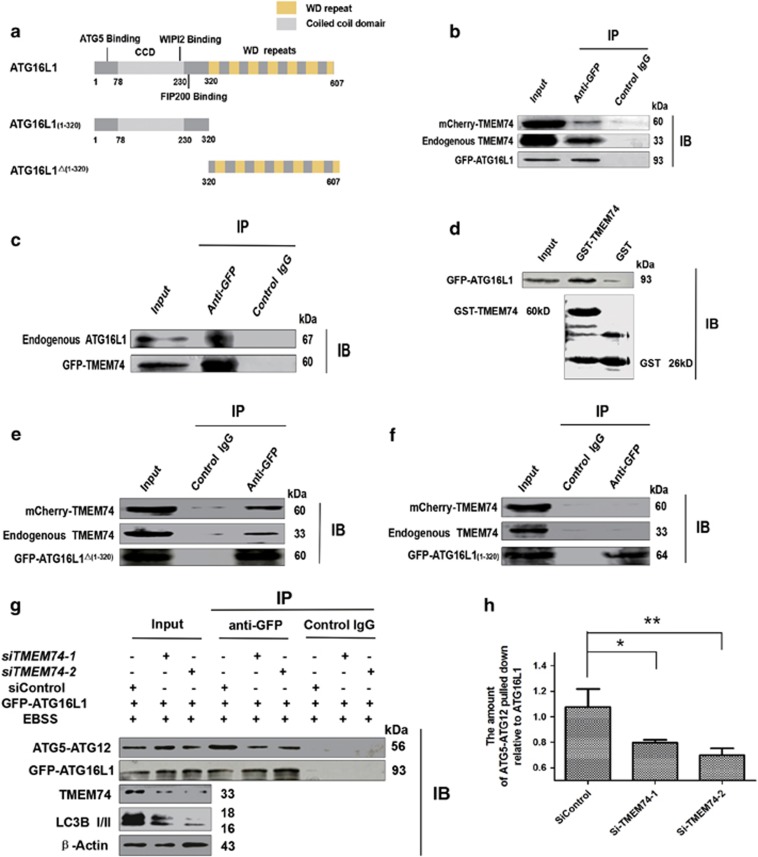
TMEM74 associates with ATG16L1 via its C-terminal and influences the interaction between ATG5 and ATG16L1. (**a**) Schematic representations of WT ATG16L1 and its mutants: ATG16L1(1–320), and ATG16L1^△(1–320)^. (**b**) HeLa cells were co-transfected with GFP-ATG16L1 and mCherry-TMEM74 for 24 h, Total cell extracts were subjected to IP using either an anti-GFP or an isotype control IgG, TMEM74 was detected in the washed beads using anti-TMEM74 IgG by western blotting. (**c**) HeLa cells were co-transfected with GFP-TMEM74 and mCherry-ATG16L1 for 24 h. Total cell extracts were subjected to IP using either an anti-GFP or an isotype control IgG, ATG16L1 was detected in the washed beads using an anti-ATG16L1 IgG by western blotting. (**d**) GST and GST-TMEM74 fusion protein immobilized on glutainione-sepharose beads were incubated with HeLa cell lysates containing GFP-ATG16L1, GFP-ATG16L1 was detected in the washed beads by western blotting. (**e**,**f**) HeLa cells were co-transfected with mCherry-TMEM74 and GFP-ATG16L1(1–320), or GFP-ATG16L1^△(1–320)^ respectively for 24 h. Total cell extracts were subjected to IP using an anti-GFP or an isotype control IgG, as indicated. TMEM74 were detected in the washed beads by western blotting. (**g**,**h**) HeLa cells were firstly treated by *siTMEM74-1*,*siTMEM74-2* or siControl for 24 h, then transfected with GFP-ATG16L1 for 24 h, meanwhile treated with EBSS for at least 8 h. Total cell extracts were subjected to IP using an anti-GFP or a non-specific control IgG, ATG5-ATG12 complex pulled down was detected in the immunoprecipitates using anti-ATG5 by western blotting. Quantification of ATG5-ATG12 pulled down relative to GFP-ATG16L1 was shown as column. Data are means±S.D. of three experiments. *P<0.05, **P<0.01

We tested the interactions of TMEM74 with several autophagy modulators. The full-length and truncated plasmids, GFP-ATG16L1, GFP-ATG16L1_(1–320)_ and GFP-ATG16L1^△(1–320)^ were constructed (Figure 2a). According to a Co-IP assay, mCherry-TMEM74 and endogenous TMEM74 were detected in GFP-ATG16L1 immunoprecipitates by an anti-GFP IgG antibody (Figure 2b). Similar to this result, endogenous ATG16L1 were also captured in the GFP-TMEM74 immunoprecipitates (Figure 2c). Next, we obtained a GST-tagged full-length TMEM74 fusion protein and performed a pull-down assay; GST-TMEM74 bound to GFP-ATG16L1 *in vitro* (Figure 2d). Thus, we identified an interaction between TMEM74 and ATG16L1.

Furthermore, GFP-ATG16L1^△(1–320)^ precipitated mCherry-TMEM74 and endogenous TMEM74 (Figure 2e), conversely, GFP-ATG16_(1-320)_ failed to bind mCherry-TMEM74 (Figure 2f), suggesting that the WD repeats domain of ATG16L1 was required for the interaction with TMEM74.

We further identified the role of TMEM74 in the ATG5-ATG12/ATG16L1 complex. Intriguingly, we observed a substantial decrease in the levels of endogenous ATG5-ATG12 in the GFP-ATG16L1 immunoprecipitates in HeLa cells transfected with *siTMEM74-1/siTMEM74-2* that had been treated with EBSS for 8 h (Figures 2g and h). ATG16L1 is essential for ATG5-ATG12 conjugation to phagophore membranes to activate the LC3 recruitment,^26^ so we hypothesized that TMEM74 interacted with ATG16L1 as a ‘catalyst’ to initiate the formation of the ATG5-ATG12/ATG16L1 complex.

### TMEM74 associates with ATG9A via the N-terminal region of ATG9A

Next, other molecules were examined whether to interact with TMEM74. Firstly, mCherry-ATG5 and mCherry-ATG5-ATG12 (overexpressed mCherry-ATG5 conjugated to endogenous ATG12) were not detected in the GFP-TMEM74 immunoprecipitates (Supplementary Figure 4SA). Similarly, GFP-TMEM74 did not precipitate mCherry-LC3B in cell lysates (Supplementary Figure 4SB). In conclusion, the results excluded the possibility that ATG5 and LC3B interact with TMEM74.

**Figure 3:**
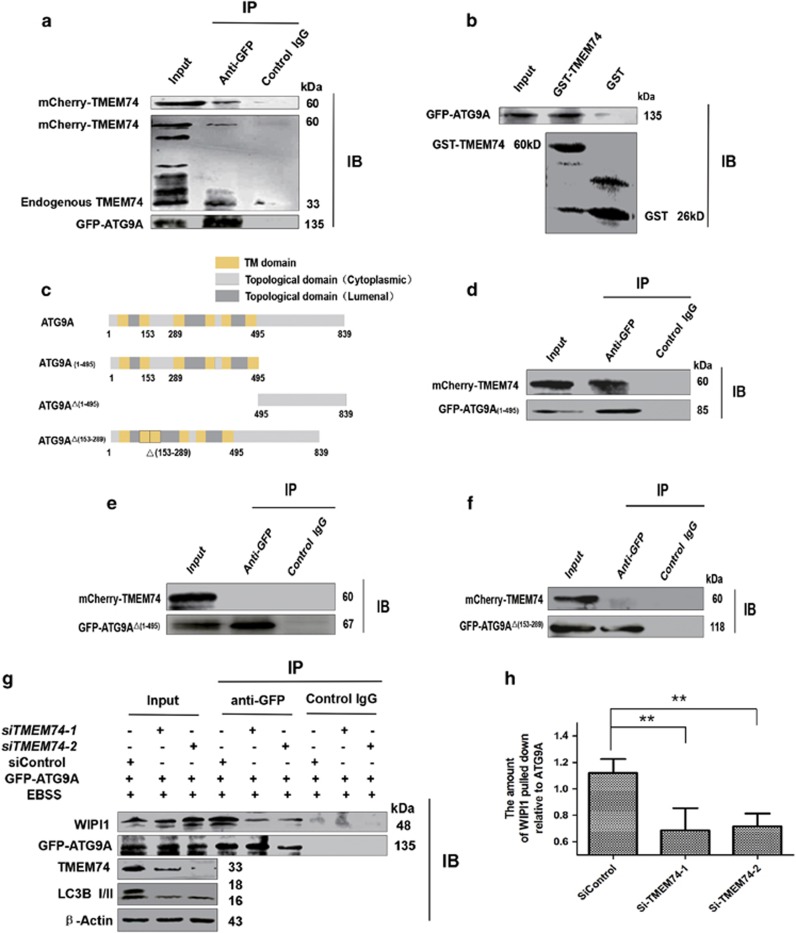
TMEM74 associates with ATG9A via its N-terminal and influences the interaction between ATG9 and WIPI1. (**a**) HeLa cells were co-transfected with GFP-ATG9A and mCherry-TMEM74 for 24 h. Total cell extracts were subjected to IP using either an anti-GFP or an isotype control IgG, TMEM74 was detected in the washed beads using anti-TMEM74 IgG by western blotting. (**b**) GST and GST-TMEM74 fusion protein immobilized on glutainione-sepharose beads were incubated with HeLa cell lysates containing GFP-ATG9A, GFP-ATG9A was detected in the washed beads using an anti-GFP IgG by western blotting. (**c**) Schematic representations of WT-ATG9A and its mutants: ATG9A(1–495), and ATG9A^△(1–495)^, and ATG9A^△(153–289)^. (**d**–**f**) HeLa cells were co-transfected with mCherry-TMEM74 and GFP-ATG9A(1–495), GFP-ATG9A^△(1–495)^, or GFP-ATG9A^△(153–289)^ respectively for 24 h. Total cell extracts were subjected to IP using an anti-GFP or an isotype control IgG, as indicated. TMEM74 was detected in the washed beads by western blotting. (**g**,**h**) HeLa cells were firstly treated by *siTMEM74-1*, *siTMEM74-2* or siControl for 24 h, then transfected with GFP-ATG9A for 24 h, meanwhile treated with EBSS for at least 8 h. Total cell extracts were subjected to IP using an anti-GFP or a non-specific control IgG, WIPI1 pulled down was detected in the immunoprecipitates using the anti-WIPI1 antibody by western blotting. Quantification of WIPI1 pulled down relative to GFP-ATG9A was shown as column. Data are means±S.D. of three experiments. *P<0.05, **P<0.01

We constructed full-length and truncated ATG9A plasmids: GFP-ATG9A, some variants in which the entire N-terminal region (ATG9A^△(1–495)^), C-terminal region (ATG9A_(1–495)_) or the longest cytoplasmic domain (CD) (ATG9A^△(153–289)^) were missing (Figure 3c). Over-expressed mCherry-TMEM74 and endogenous TMEM74 were observed by western blotting and showed that TMEM74 interacted with ATG9A (Figure 3a). A subsequent GST pulldown assay revealed a direct interaction between TMEM74 and ATG9A, and GST-TMEM74 fusion proteins were colocalized with overexpressed GFP-ATG9A in HeLa cells (Figure 3b).

Furthermore, only GFP-ATG9_(1–495)_ precipitated mCherry-TMEM74 in cell lysates, indicating that the N-terminal region of ATG9A binds TMEM74 and the CD is required for the interaction, as the deletion of the CD disrupted the association (Figures 3d–f).

Atg9 recycling for membranous materials trafficking between PAS and peripheral compartments like TGN (*trans*-Golgi networks), endosomes hinges on Atg18 by the interaction.^20, 27, 28^ We then assessed the effect of TMEM74 on ATG9A binding to WIPI1, the human homolog of Atg18. Decreased levels of endogenous WIPI1 were detected in the GFP-ATG9A immunoprecipitates from HeLa cells transfected with *siTMEM74-1* and *siTMEM74-2* compared with cells transfected with the siControl (Figures 3g and h). Thus, TMEM74 may regulate the association of ATG9A with WIPI1 and ATG9A trafficking.

### TMEM74-triggered autophagy is associated with the ATG5-ATG12/ATG16L1 complex and acts downstream of ULK1 and the BECN1/PI3KC3 complex

We continued to examine the molecules that function in TMEM74-induced autophagy and at which steps autophagy induction is obstructed in TMEM74-overexpressing cells.

**Figure 4:**
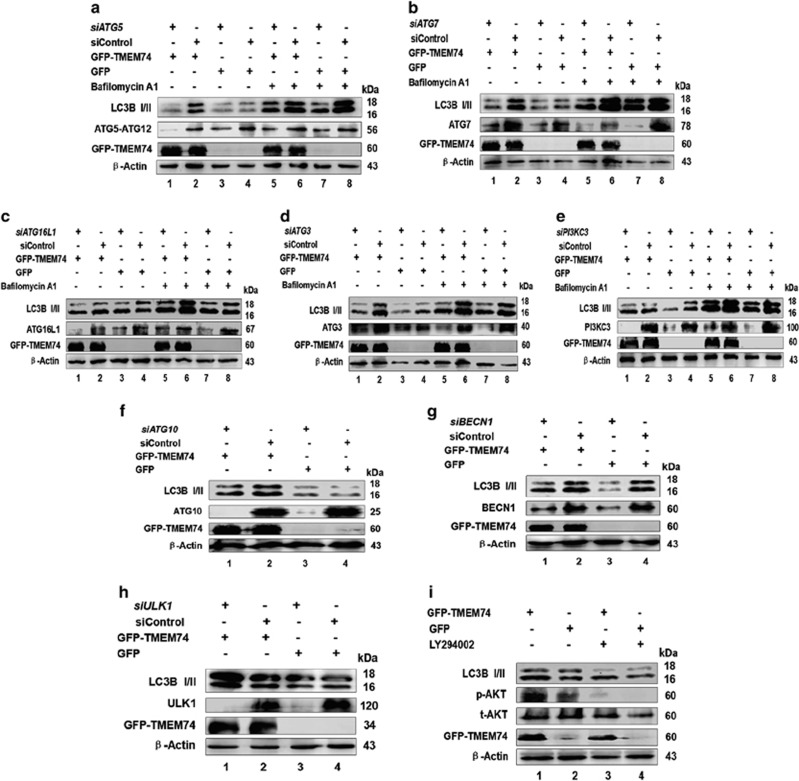
TMEM74-induced autophagy is associated with ATG5-ATG12 complex, independent with BECN1-PIK3C3 complex. HeLa cells were firstly treated with the indicated siRNAs (*siATG5* (**a**), *siATG7* (**b**), *siATG16L1* (**c**), *siATG3* (**d**), *siPI3KC3* (**e**), *siATG10* (**f**), *siBECN1* (**g**), *siULK1* (**h**)) for 24 h, then transfected with GFP-TMEM74 or GFP for 24 h,meanwhile treated with or without bafilomycin A1 (100 nM) for at least 12 h. The levels of LC3B-II were detected by western blotting. (**i**) HeLa cells were transfected with GFP-TMEM74 or GFP (control) for 24 h respectively, treated with or without LY294002 (10 *μ*M) for at least 8 h. The levels of LC3B-II were detected by western blotting

Firstly, we focused on the two ubiquitin-like complexes, ATG5- ATG12/ATG16L1 and LC3B/PE, as well as ATG7, ATG10 and ATG3. At this point, we silenced the expression of the *ATG5*, *ATG7*, *ATG16L1*, *ATG3* and *ATG10* in TMEM74-overexpressing HeLa cells. According to western blots, knockdown of *ATG5*, *ATG7*, *ATG16L1 ATG3* and *ATG10* (without bafilomycin A1 treatment) reversed the LC3B-II accumulation induced by TMEM74 overexpression (the gap between lane 1 and lane 3 *versus* that between lane 2 and lane 4) (Figures 4a–d). Thus, TMEM74-triggered autophagy relies on the formation of the ATG5-ATG12/ATG16 and LC3B/PE complexes.

We also examined the molecules upstream of autophagy: BECN1, PI3KC3 and ULK1, and performed similar experiments. Knockdown of *PI3KC3* (with bafilomycin A1 treatment), *BECN1*, *ULK1* failed to repress the increase in the endogenous LC3B-II levels stimulated by TMEM74 overexpression (the gap between lane 1 and lane 3 *versus* that between lane 2 and lane 4) (Figures 4e–h), indicating that TMEM74 induced a BECN1/PI3KC3- and ULK1-independent autophagy pathway. Similarly, the TMEM74-induced increase in the LC3B-II levels was not inhibited by the LY294002 (a PI3K inhibitor) treatment (Figure 4i). The results supported that TMEM74-induced autophagy acts downstream BECN1/PI3KC3 complex and ULK1.

### TMEM74-induced autophagy positively regulates the signaling pathways related to cell survival

We next investigated the signaling pathways involved in both autophagy and cell survival. The PI3KC1 (PI3K in the following)/AKT/mTOR pathway that functions in response to extrinsic stimuli were examined in our study. PI3K/AKT/mTOR pathway transducts the signals from receptor tyrosine kinases and G protein-coupled receptors for its activation to phosphorylate the PI(4,5)P2 to PI(3,4,5)P3, then PI3P recruits substrates containing PH domain to membranes. AKT phosphorylation contributes to mTOR activation(at Ser2448), reversely inhibits autophagy.^29, 30^ Additionally, GSK-3*β*can also be phosphorylated by AKT(at Ser9), thus inhibits glycogen synthesis. AMPK(a heterotrimer containing *α*, *β*, *γ*, subunits) is a energy sensor whose activity is dependent of the AMP/ATP or ADP/ATP ratio.^31^ P38-MAPK, as a member of mitogen-activated protein kinases (MAPKs), can be strongly activated by inflammatory cytokines, growth factors and many other stimuli, which has been evidenced to play a dual role in different tumor types.^32^

**Figure 5:**
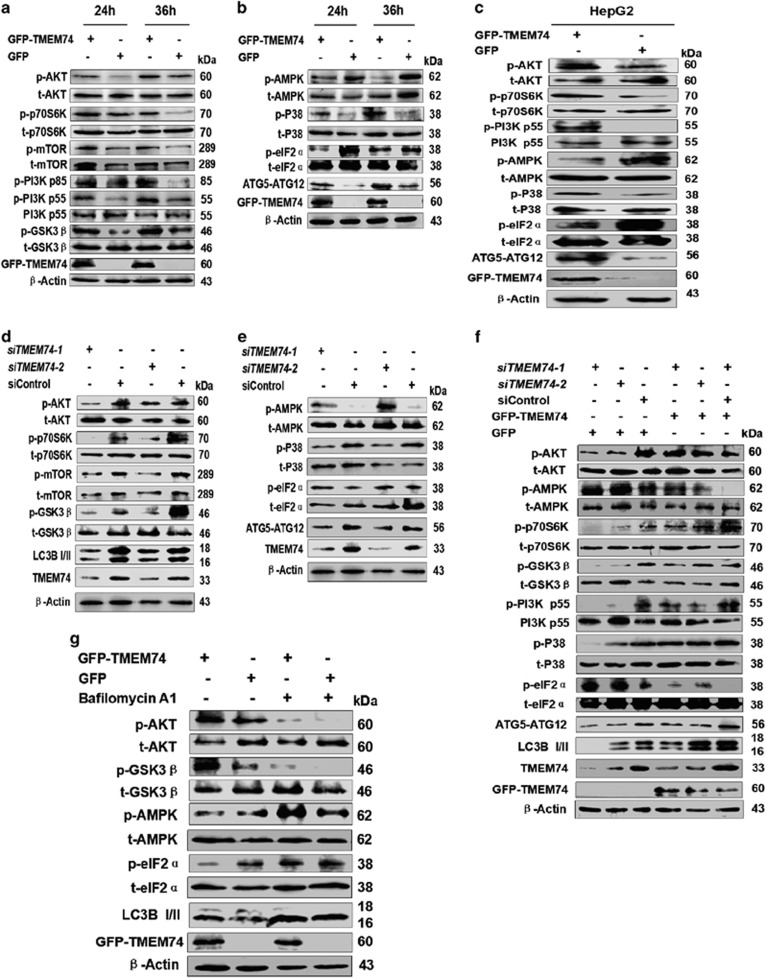
TMEM74 positively influences the signal pathways relevant to cell survival. (**a**,**b**) Western blotting analysis of total and phosphorylation levels of AKT (Ser473), p70S6K (Thr389), mTOR (Ser2448), PI3K p85 (Tyr458), PI3K p55 (Tyr199), GSK-3*β* (Ser9), AMPK*α* (Thr172), p38 MAPK (Thr180/Tyr182), eIF2*α* (Ser51), and ATG5-ATG12 complex in HeLa cells transfected with GFP-TMEM74 or GFP (control) for 24 and 36 h were shown. (**c**) Western blotting analysis of total and phosphorylation levels of AKT (Ser473), p70S6K (Thr389), PI3K p55 (Tyr199), AMPK*α* (Thr172), p38 MAPK (Thr180/Tyr182), eIF2*α* (Ser51), and ATG5-ATG12 complex in HepG2 cells transfected with GFP-TMEM74 or GFP (control) for 24 h was shown. (**d**,**e**) Western blotting analysis of total and phosphorylation levels of AKT (Ser473), p70S6K (Thr389), mTOR (Ser2448), GSK-3*β* (Ser9), AMPK*α* (Thr172), p38 MAPK (Thr180/Tyr182), eIF2*α* (Ser51), ATG5-ATG12 complex and LC3B-II in HeLa cells transfected with *siTMEM74-1*, *siTMEM74-2* or siControl for 48 h were shown. (**f**) HeLa cells were firstly treated with *siTMEM74-1*, *siTMEM74-2* or siControl for 24 h, then transfected with GFP-TMEM74 or GFP(control) for 24 h. Cell extracts were analyzed by western blotting as indicated. (**g**) HeLa cells were transfected with GFP-TMEM74 or GFP (control), and treated with bafilomycin.A1 (100 nM). Cell extracts were analyzed by western blotting as indicated

We performed experiments to explore the alterations of signal pathways engendered by TMEM74. Intriguingly, the PI3K/AKT/mTOR pathway was activated, as supported by the increased levels of phosphorylated AKT (Ser473), PI3K p85 (Tyr458), PI3K p55 (Tyr199), mTOR (Ser2448), p70S6K (Thr389) and GSK3*β* (Ser9) (Figure 5a). In contrast, decreased levels of phosphorylated AMPK*α* (Thr172), and eIF2*α* (Ser51) were observed, elevated levels of phosphorylated p38-MAPK were also observed (Figure 5b). Similar results were obtained in HepG2 cells (Figure 5c).

The activation of the signaling molecules significantly decreased in the *TMEM74-*silenced HeLa cells (Figures 5d and e). A rescue experiment showed that the decrease in the activity of signaling pathways induced by *TMEM74* knockdown was reversed by TMEM74 overexpression (Figure 5f). In addition, bafilomycin A1 counteracted the effect elicited by TMEM74, indicating that TMEM74-induced autophagy alters the signaling pathways (Figure 5g). To make it robust, we observed the changes of AKT phosphorylation in the depletion of *ATG5*, *ATG16L1*, *ATG7*, *ATG3*, *ATG10*, *BECN1* and *PI3KC3*, and results showed the absence of the *ATG5*, *ATG16L1*, *ATG7*, *ATG3*, and *ATG10* effectively inhibited the changes resulting from TMEM74 overexpression(Supplementary Figures 5SA–E). However, the absence of *BECN1* and *PI3KC3* manifested no impact on the AKT phosphorylation (Supplementary Figures 5SF and G), which indicates that TMEM74-induced autophagy may act as the prominent cause to alter the signal pathways. Notably, the ATG5-ATG12 complex was upregulated by TMEM74 overexpression, indicating that TMEM74 promotes the formation of the ATG5-ATG12 complex, consistent with the results shown in Figures 5b.

### TMEM74 positively regulates tumor cell survival

**Figure 6:**
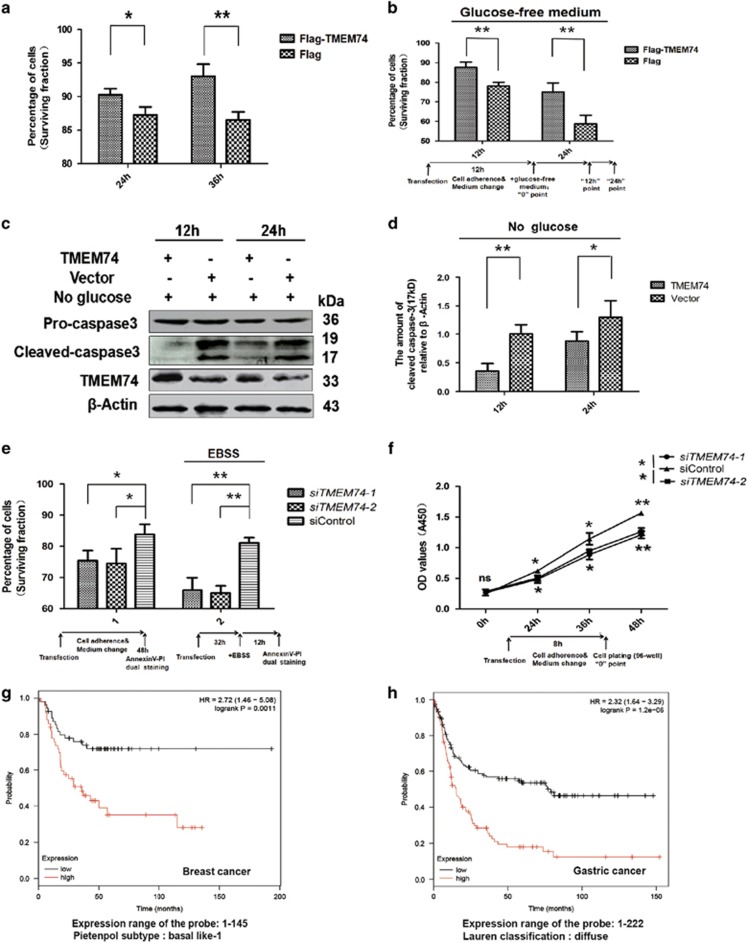
TMEM74 positively influences cell proliferation and survival. (**a**,**b**) HeLa cells were transfected with Flag-TMEM74 or Flag (control) respectively for 24 and 36 h, cultured with or without glucose-free medium for 12 or 24 h, the apoptotic cells were measured with AnnexinV-PI dual staining followed by flow cytometry. Data are means±S.D. of three experiments. *P<0.05, **P<0.01. (**c**,**d**) HeLa cells were transfected TMEM74 or control plasmids for 12 h, then cultured with glucose-free medium for 12 or 24 h, the levels of cleaved caspase-3 were detected by western blotting. Quantification of cleaved caspase-3(17kD) levels relative to *β*-Actin was shown as column. Data are means±S.D. of three experiments. *P<0.05, **P<0.01. (**e**) HeLa cells were transfected with *siTMEM74-1*, *siTMEM74-2* or siControl for 48 h, treated with or without EBSS for at least 12 h, the apoptotic cells were measured with AnnexinV-PI dual staining followed by flow cytometry. Data are means±S.D. of three experiments. Data are means±S.D. of three replicates. *P<0.05, **P<0.01. (**f**) HeLa cells were transfected with *siTMEM74-1*, *siTMEM74-2* or siControl, then monitored the cell proliferation by CCK-8 assay (Cell counting Kit-8). Data are means±S.D. of three replicates. *P<0.05, **P<0.01. (**g**,**h**) The analysis of the surviving periods of the indicated cancer patients samples belonging to specific subtype histology type or classification in high and low expression of TMEM74 was shown from the KM plotter

After the confirmation of alternation of signal pathways, we next assessed whether TMEM74 promotes tumor cell survival. Firstly, experiments were performed in HeLa cells. Based on flow cytometry analysis, TMEM74 increased the percentage of surviving cells at two time points (Figure 6a), and a similar result was obtained using CCK-8 (cell counting kit-8) assays (Supplementary Figure 6SA). When HeLa cells were treated with bafilomycin A1, the inhibitor of autophagosome formation, the fraction of surviving HeLa cells was reduced in the CCK-8 assay (Supplementary Figure 6SB). Moreover, fewer TMEM74-overexpressing HeLa cells underwent cell death in response to metabolic stress, as assessed by flow cytometry analysis (Figure 6b). Simultaneously, the results of the CCK-8 assay in HeLa cells treated with glucose-free medium and etoposide (an anti-cancer drug) were consistent with the former flow cytometry analysis (Supplementary Figures 6SC and D). Same experiments were repeated in glucose-starved HepG2, U2OS and 786-O cells and similar results were obtained as assessed by flow cytometry (Supplementary Figures 7SA–F). Furthermore, the western blots showed decreased cleaved caspase-3 levels in the TMEM74-transfected HeLa cells under metabolic stress (Figures 6c and d).

Reciprocally, *TMEM74* knockdown led to the opposite cell viability effects on normal or EBSS starvation-treated cells (Figures 6e and f), suggesting the lack of resistance to starvation was ascribed to the *TMEM74* knockdown-mediated loss of autophagy.

According to an analysis using KM plotter, it was found that the high expression of TMEM74 correlates tightly with the survival periods in the breast cancer and gastric cancer. Considering the factors of the cancer-like grade (stage), and subtypes (histology or classification), high expression of TMEM74 in clinical samples significantly reduces relapse-free survival in breast cancer belonging to basal-like 1 Pietenpol subtype (Figure 6g), Besides, the grades 1 and 2 breast cancer samples also exhibits the same results(Supplementary Figures 8SB and C). Meanwhile, the gastric cancer belonging to diffuse types (Figure 6h), intestinal and mixed types (Supplementary Figures 8SD and E), their first progression survival periods also decline at the high expression of TMEM74. Additionally, the first progression survival of stage 3 gastric cancer likewise associates with the expression of TMEM74 (Supplementary Figure 8SA). In conclusion, the results indicate tumor progression associates tightly with high TMEM74 expression. Thus, TMEM74 may be a target for cancer therapy or a prognostic factor.

### TMEM74-induced autophagy contributes to tumor cell survival

**Figure 7:**
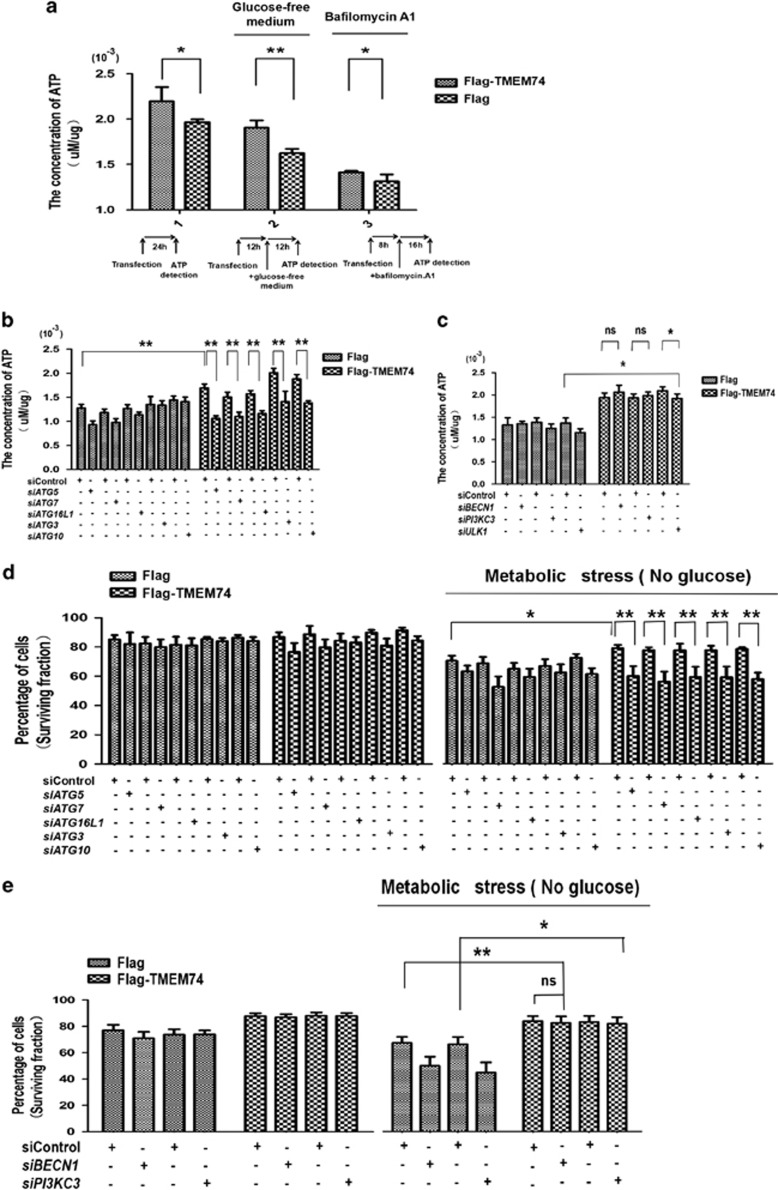
TMEM74-induced autophagy increases ATP levels and favors cell survival under metabolic stress. (**a**) Cytosolic ATP levels in HeLa cells transfected with Flag-TMEM74 or Flag (control) for 24 h were measured in the absence of glucose or under bafilomycin.A1 (100 nM) treatment by ATP chemiluminescence detection kit. Data are means±S.D. of five replicates. *P<0.05, **P<0.01. (**b**,**c**) HeLa cells were firstly treated with the indicated siRNAs for 24 h (*siATG5*, *siATG7*, *siATG16L1*, *siATG3*, *siATG10*, *siBECN1*, *siPIK3C3*, *siULK*, siControl), then transfected with Flag-TMEM74 or Flag (control) for 24 h. The levels of cytosolic ATP were measured by ATP detection kit. Data are means±S.D. of three replicates. *P<0.05, **P<0.01. (**d**,**e**) HeLa cells were firstly treated with the indicated siRNAs for 24 h (*siATG5*, *siATG7*, *siATG16L1*, *siATG3*, *siATG10*, *siBECN1*, *siPIK3C3*, *siULK*, siControl), then transfected with Flag-TMEM74 or Flag (control) for 24 h, and subjected to glucose-free medium for at least 12 h. The apoptotic cells were measured with AnnexinV-PI dual staining followed by flow cytometry. Data are means±S.D. of three replicates. *P<0.05, **P<0.01

Next, we determined whether TMEM74-induced autophagy is credited with tumor cell survival. We utilized an ATP chemiluminescence detection kit to directly measure the ATP levels. The cytosolic ATP levels were elevated in TMEM74-overexpressing HeLa cells under normal conditions, energy stress or even bafilomycin A1 treatment (Figure 7a). However, the capacity of TMEM74-overexpressing cells to maintain high ATP levels was compromised by the depletion of *ATG5*, *ATG7*, *ATG16L1*, *ATG3* and *ATG10* (Figure 7b), but not the absence of *BECN1*, *PI3KC3* or *ULK1* (Figure 7c). These results suggest that TMEM74-induced autophagy allows tumor cells to maintain high ATP levels.

Regarding tumor cell survival, the resistance to metabolic stress induced by TMEM74 overexpression was counteracted by *ATG5*, *ATG7*, *ATG16L1*, *ATG3* and *ATG10* deficiencies (Figure 7d) but not the *BECN1* and *ULK1*(Figure 7e). The fractions of surviving *si-BECN1-* and *siPI3KC3*-transfected cells overexpressing TMEM74 were larger than the fractions of normal cells. Based on these results, TMEM74-induced autophagy promotes tumor cell survival.

### Autophagy regulates the expression of the TMEM74 mRNA and protein

**Figure 8:**
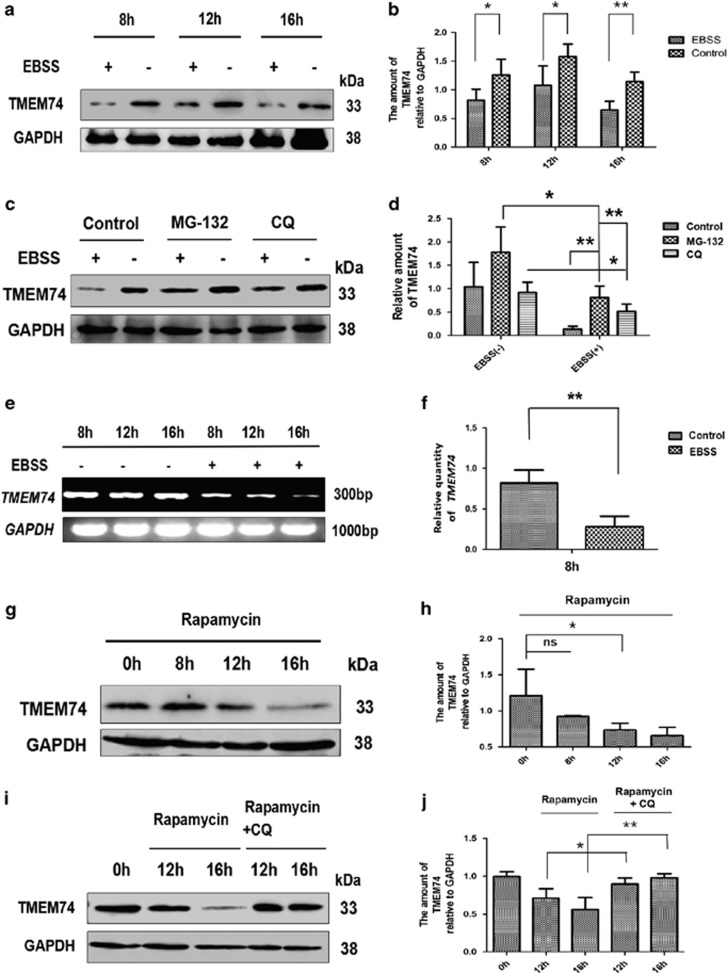
The expression of TMEM74 in the autophagic process. (**a**,**b**) Western blotting analysis of endogenous TMEM74 levels in HeLa cells treated by EBSS (8, 12, 16 h). Quantification of TMEM74 levels relative to GAPDH was shown as column. Data are means±S.D. of three experiments. *P<0.05, **P<0.01. (**c**,**d**) Western blotting analysis of endogenous TMEM74 levels in HeLa cells treated by EBSS for 8 h with/without MG-132 (10 *μ*M) or CQ (25 *μ*M). Quantification of TMEM74 levels relative to GAPDH was shown as column. Data are means±S.D. of three experiments. *P<0.05, **P<0.01. (**e**,**f**) HeLa cells were treated with EBSS (8, 12, 16 h). the levels of TMEM74 were detected by RT-PCR and Q-PCR, respectively. (**g**,**i**) Western blotting analysis of endogenous TMEM74 levels in HeLa cells treated by rapamycin (500 nM) for 8,12 and 16 h with/without CQ (25 *μ*M) for 12 and 16 h. (**h**,**j**) Quantification of TMEM74 levels relative to GAPDH was shown as column. Data are means±S.D. of three experiments. *P<0.05, **P<0.01

It was then assessed why TMEM74-triggered autophagy promotes tumor cell survival and determined the differences from other forms of ‘autophagy’, such as upstream signal-mediated autophagy. Therefore, we detected endogenous TMEM74 expression during common autophagic processes. Based on western blotting, TMEM74 was downregulated in EBSS- or rapamycin-treated HeLa cells in a time-dependent manner (Figures 8a). We treated the EBSS-treated cells with MG-132 and CQ to determine the mechanism by which the TMEM74 levels were reduced. Both MG-132 (a proteasome inhibitor) and CQ (an autolysosome inhibitor) partially reversed the downregulation of TMEM74 expression (Figures 8c and d). In addition, the expression of the TMEM74 mRNA was detected by RT-PCR and Q-PCR in the HeLa cells subjected to starvation, and TMEM74 expression was reduced (Figures 8e and f). However, CQ primarily compromised the rapamycin-induced downregulation of TMEM74, indicating that rapamycin-mediated degradation of TMEM74 mainly relies on autolysosomes (Figures 8i and j). Thus, the downregulation of TMEM74 expression was attributed to proteasomes, autophagy and gene regulation.

## Discussion

TMEM74, which has been shown to stimulate cell autophagy, was characterized in our lab.^22^ However, its detailed mechanisms of action were not illuminated. In this study, firstly, TMEM74 was shown to increase autophagic flux in diverse tumor cell lines and interact with ATG16L1 and ATG9A to induce autophagy. TMEM74-induced autophagy also elicits a positive effect on tumor cell survival, high expression of TMEM74 can be considered as a risk factor, which shortens the surviving progression of cancer patients according to the clinical database analysis, so it may be a target for cancer therapy or a prognostic factor.

The following discussion is separated into two parts that discuss the mechanism underlying TMEM74-induced autophagy and the hypothesis to explain why TMEM74-induced autophagy has a beneficial effect on tumor cell survival.

### The mechanism by which TMEM74 induces a novel autophagic pathway independent of PI3K activity

Phagophores generally originate from a PAS (pre-autophagosomal structure) in yeast, or nucleate at a complex membranous structure termed the omegasome in higher eukaryotes.^33^ Omegasomes typically occur at ER-associated membranes, and phagophore nucleation has recently been shown to occur at ER-mitochondria contact sites.^34^ In addition, the outer mitochondrial membrane provides phagophores with membranous components. Moreover, the membranous components required for phagophore expansion can be obtained from ERES(ER exit sites),^35^ ERGIC (the ER–Golgi intermediate compartment),^36^ the Golgi, the plasma membranes or recycling endosomes.^37, 38^ However, the essential reason why phagophore nucleation requires key molecules such as PI3KC3 is that most ATG molecules lack typical membrane-binding motifs and must rely on other molecules to bind to the membranes. Based on the localization of TMEM74 in the ER and mitochondria and the TM domains of TMEM74, TMEM74 may be regarded as another key phagophore nucleation molecule or a substitute to recruit other ATG proteins like a platform.

When TMEM74 anchors to the source membranes, it recruits ATG16L1 and then promotes the formation of the ATG5-ATG12/ATG16L1 complex via ATG7 and ATG10. Next, ATG3-LC3 intermediates activated by ATG7 are recruited to the membranes via the interaction between ATG3 and ATG12, which brings LC3 in proximity to PE in the membranes, leading to subsequent lipidation.^26, 39^

Additionally, the TMEM74 interaction with ATG9A may tether the vesicles to phagophores for their expansion. ATG9A recycling between the PAS and peripheral structures is required for the transport of membrane materials. The trafficking of ATG9A from the phagophore precursors requires the peripheral protein Atg18. Thus, according to our results, the interactions between TMEM74 and ATG9A may be required for anterograde transport, and the TMEM74-mediated interaction between ATG9A and WIPI1 (the homolog of yeast Atg18) may contribute to retrograde transport.

In conclusion, TMEM74 stimulates autophagy by directly triggering the formation of autophagosomes, bypassing the upstream signal pathways including the initial stage mediated by PI3P. However, besides the nucleation process, TMEM74 can also be considered as a fundamental component to be responsible for the subsequent stages including membranous expansion and maturation supported by the results Supplementary Figure 2S. Thereby, TMEM74 can be reasonably regarded as a critical initiator and promoter in the course of autophagosome formation for common autophagy.

### Model of the hypothesized ‘self-regulatory’ loop that controls autophagy levels

The relationship between autophagy and cell death or survival remains controversial. Excessive autophagy definitely induces cell death, such as EBSS-induced autophagy. However, autophagy itself leads to the turnover of cellular material and produces energy for cell metabolism.^40^ Consistent with the results that TMEM74-induced autophagy promotes tumor cell survival, TMEM74 is also downregulated during the autophagic process. We constructed a ‘self-regulatory loop’ model to explain the phenomenon.

Considering that TMEM74-triggered autophagy can be maintained at a proper level by reducing the amount of this inducer via diverse pathways, including autolysosome digestion, which forms a self-regulatory loop that differs from other common types of ‘autophagy’ induced by extracellular or intracellular factors. So the TMEM74-induced autophagy is positive for cell viability. The controllability and feedback characteristics are crucial features of TMEM74-induced autophagy. Thus, maximal positive effects of autophagy on tumor cell survival emerge.

In summary, TMEM74-induced autophagy may represent a novel autophagy pathway and a new model to substantiate the relationship between autophagy and tumor cell survival. Further studies examining the properties of TMEM74 or similar molecules to understand autophagy will be important and may even represent targets for cancer therapy. The diagram of what are mentioned in discussion is shown in Supplementary Figure 9S.

## Materials and methods

### Antibodies and reagents

Polyclonal antibodies against TMEM74 produced and purified by our lab; anti-caspase-3 (CST, Boston, USA, 9665S); anti-Actin (Tianjin Sungene Biotech, Tianjin, China, KM9001); anti-Flag (Tianjin Sungene Biotech, KM8002); anti-Myc (Tianjin Sungene Biotech, KM8003); Anti-GST (MultiSciences, Hangzhou, China, ab004-040); anti-GFP (Abcam, Cambridge, UK, ab1218), anti-GFP (Sigma Aldrich, Darmstadt, German, SAB4301138); Anti-GAPDH (Tianjin Sungene LK9002); anti-ATG5 (CST, 4445); anti-ATG7 (CST,4445); anti-ATG16L1 (CST,4445); anti-ATG3 (CST,4445); anti-Beclin1 (CST,4445); anti-ULK1 (Abcam, ab120243); anti-Apg10 (Abcam, ab124711); anti-PI3KC3 (Abcam, ab124905); anti-p62 (CST, 5114); anti-p-AKT (CST,4051S); anti-AKT (CST, 4691S); anti-p-PI3K(CST, 4228P); anti-PI3K p55 (CST, 11889S); anti-p-p70S6K (CST,9234S); anti-p70S6K (CST, 2708S); anti-p-mTOR (CST, 2971S); anti-mTOR (CST,2983S); anti-p-p38MAPK (CST, 4511S); anti-p38MAPK (CST, 8690S); anti-p-AMPK*α* (CST, 2531S); anti-AMPK*α* (CST, 2532S); anti-p-GSK3*β* (CST, 9323S); anti-GSK3*β* (CST, 12456S); anti-p-eIF2*α* (CST, 3398P); anti-eIF2*α* (CST, 5324S); anti-WIPI1 (CST, 12124S); anti-LC3B (Sigma Aldrich, L7543); HRP-conjugated goat anti-rabbit secondary antibody (Biodragon, Beijing, China, BF03008); HRP-conjugated goat anti-mouse secondary antibody (Biodragon, BF03001); Bafilomycin.A1 (Santa Cruz Biotechnology, Santa Cruz, CA, USA, sc-201550A); Rabbit control IgG (Biodragon, BF01001); Mouse control IgG (Biodragon, BF01116); BL21 competent cells (Solarbio, Beijing, China, C1400-10); CCK-8 (DOJINDO, Shanghai, China, CK04); AnnexinV-PI apoptosis detection kit (DOJINDO, AD10); LY294002 (Abcam, ab120243); ATP detection kit (Beyotime Biotechnology, Nanjing, China, S0026); MG-132 (Abcam, ab146600); CQ (Sigma Aldrich, C6628); Rapamycin (Beyotime Biotechnology, S1842); No-glucose DMEM medium (BasalMedia (Yuanpei Biotech), Shanghai, China, L160); EBSS (Sigma Aldrich, E7510); Lipofectamine 3000 transfection reagent (Invitrogen, Carlsbad, CA, USA, L3000015).

### Plasmid construction and siRNA

Flag-TMEM74, GFP-TMEM74, mCherry-TMEM74, TMEM74(no label), GST-TMEM74, GFP-LC3B,RFP-LC3B plasmids were generated in our laboratory, GFP-ATG16L1, GFP-ATG5, GFP-ATG9A, GFP-DFCP1, GFP-STX17, mCherry-LC3B and GFP-BECN1 plasmids were kindly provided by Yingyu Chen (Peking University, Beijing, China). Based on these plasmids, we constructed the following plasmids and truncated plasmids: mCherry-ATG5, mCherry-ATG16L1, GFP-ATG16L1_(1–320)_, GFP-ATG16L1^△(1–320)^, GFP-ATG9A^△(1–495)^, GFP-AT9A_(1–495)_ and GFP-ATG9A^△(153–289)^; additionally, the following plasmids expressing truncated TMEM74 were also constructed: GFP-TMEM74^△^, TMEM74^△^, mCherry-ER, mCherry-Mito and mCherry-LAMP1 plasmids were constructed by Polepolar Biotechnology. All plasmids were confirmed by DNA sequences.

Double-stranded siRNAs against the indicated genes were designed, and chemically synthesized by Genechem corporation (Shanghai China), which were listed in Table 1.

### Cell culture, transfections and treatments

HeLa cells, HepG2 cells, 786-O cells or U2OS cells were cultured in RPMI 1640 medium (Invitrogen 22400-089) or DMEM medium (Invitrogen, 12800-017) supplemented with 10% fetal bovine serum and maintained at 37 °C in a humidified chamber with 5% CO_2_. Cells were transfected with plasmids (10–18 *μ*g) or siRNAs using the electroporation machine (BTX,ECM630) when cell concentration was regulated at 2–3 × 10^6^/400 *μ*l. The condition of electroporation was 123–126 V, 20 ms/pulse, or using lipofectamine 3000 transfection reagent (Invitrogen, L3000015) according to the manufacturer’s instruction. Cell autophagy was induced by rapamycin (500 nM). Autolysosomes formation inhibition was achieved with bafilomycin.A1 (100 nM), which can block the fusion of autophagosomes and lysosomes, and the inhibition of autophagic substrates digestion was achieved by CQ (25 mM). Proteasomes inhibition was achieved by MG-132 (10 *μ*M), and PI3K activity inhibition was achieved by LY294002 (10 *μ*M).

### Fluorescence and confocal microscopy

HeLa cells, hepG2 cells or 786 cells co-transfected with the indicated plasmids or siRNAs were cultured in confocal dishes and treated as indicated in the experiments, fixed with 4% paraformaldehyde and permeabilized with 0.2% Triton X-100 (Beyotime, ST795). Morphological alterations in the cells were observed and documented with an Olympus FluoView FV1000 Confocal Microscope (Olympus, Melville, NY, USA).

### Flow cytometry analysis

Treated cells were trypsinized, washed with PBS and resuspended in 100 *μ*l binding buffer. Then added FITC-AnnexinV (10 *μ*l) to the cell suspension, After incubation for 20 min at 4 °C away from light, propidium iodide (PI) was added to 1 *μ*g/ml, and samples were immediately analyzed on a FACSCalibur flow cytometer (Becton Dickinson, Franklin lake, NJ, USA).

### Western blot and IP

For the western blotting analysis, treated cells were collected and disrupted in RIPA Lysis Buffer containing protease inhibitors (Roche Diagnostics, Berlin, Germany, 04693116001) then analyzed with polyacrylamide gel electrophoresis and the binds were visualized using an ImageQuant LAS 500(GE Healthcare, Glattbrugg, Switzerland).

For the IP analysis, treated cells were collected and disrupted in IP Lysis Buffer containing protease inhibitors, total cell extracts (1 mg per sample) were mixed with precleared protein G sepharose TM Fast Flow (GE Healthcare, 17-0618-01) and appropriate antibodies, followed by incubation for 4 h at 4 °C. The beads were collected by centrifugation, washed five times using washing buffer (50 mM Tris, pH 8.0, 150 mM NaCl, 0.4% NP-40, and 5 mM MgCl_2_), resuspended in 2 × SDS loading buffer and then subjected to western blotting as described previously.

### GST pull down assay

Recombinant GST or GST-TMEM74 fusion proteins were expressed in *Escherichia coli* strain BL21 (DE3) and purified. Equal amounts of these proteins were mixed with whole-cell lysates extracted from plasmid transfected cells and glutathione-Sepharose 4B (GE Healthcare, 17-0756-01) for 4 h at 4 °C. After five washes, the beads were resuspended in 2 × SDS loading buffer and analyzed by western blotting.

### Reverse transcription PCR and Q-PCR

Total RNA samples were extracted from cells with the TRIzol reagent (Invitrogen, 15596-026). RT-PCR was performed using the ThermoScript RT-PCR System (Invitrogen, 11146-024). Primers used for amplifying TMEM74 were 5′-TCCAGACATCTAAACATCTAGACAGG-3′ (forward) and 5′-CCCCAAACAGCAATAACAAGA-3′ (reverse) for Q-PCR, 5′-TTTCTCCGGGAACCGTGC-3′(forward) and 5′-TTGGGACCACTCATTTGGATTT-3′(reverse) for RT-PCR. Primers used for amplifying GAPDH were 5′-GACCACAGTCCATGCCATCAC-3′(forward) and 5′-TCCACCACCCTGTTGCTGTAG-3′(reverse)

### ATP detection assay

First of all, we acquired the standard curve with ATP standard solution, next treated cells were lysated with the lysate buffer at 4 °C, we collected the supernatant and averaged it into 96-well black plate (20 *μ*l/well), then added the ATP detection work solution (prepared by the ATP detection solution and ATP detection dilute solution with the ratio of 1:9) and mixed them rapidly, at least 2S later at room temperature, the RLU values could be measured by luminometer. ATP concentration values were converted with the ATP standard curve.

### Statistical analysis

Results are presented as the mean±S.D. Differences between groups were analyzed using the Student’s *t*-test for continuous variables. Statistical significance in this study was set at *P*<0.05. All reported *P*-values are two sided. All analyses were performed with GraphPad Prism 5.

## Publisher’s Note

Springer Nature remains neutral with regard to jurisdictional claims in published maps and institutional affiliations.

## Figures and Tables

**Table 1:** The sequence of siRNAs used in this study

*TMEM74* siRNA-1	5′-GGAGGAUGAUACAAGUUCA-3′
*TMEM74* siRNA-2	5′-GAGGCUGCCAUAUCUUUGA-3′
*ATG5* siRNA	5′-GCAACUCUGGAUGGGAUUG-3′
*ATG7* siRNA	5′-CAGUGGAUCUAAAUCUCAAACUGAU-3′
*ATG16L1* siRNA	5′-CCCGUGAUGACUUGCUAAA-3′
*ATG3* siRNA	5′-CCCAGAAGAGUUUGUGGCAGCUGGA-3′
*ATG10* siRNA	5′-GGAGUUCAUGAGUGCUAUA-3′
*BECN1* siRNA	5′-GCUGCCGUUAUACUGUUCU-3′
*PI3KC3* siRNA	5′-GUGUGAUGAUAAGGAAUAU-3′
*ULK1* siRNA	5′-AUCGAGAACGUCACCAAGU-3′
Control siRNA	5′-UUCUCCGAACGUGUCACGU-3′
