# Supplementary figures and images for: TMEM74 promotes tumor cell survival by inducing autophagy via interactions with ATG16L1 and ATG9A
Source: Cell Death Dis. 2017 Aug 31;8(8):e3031–. doi: 10.1038/cddis.2017.370 (PMC5596558; doi:10.1038/cddis.2017.370)

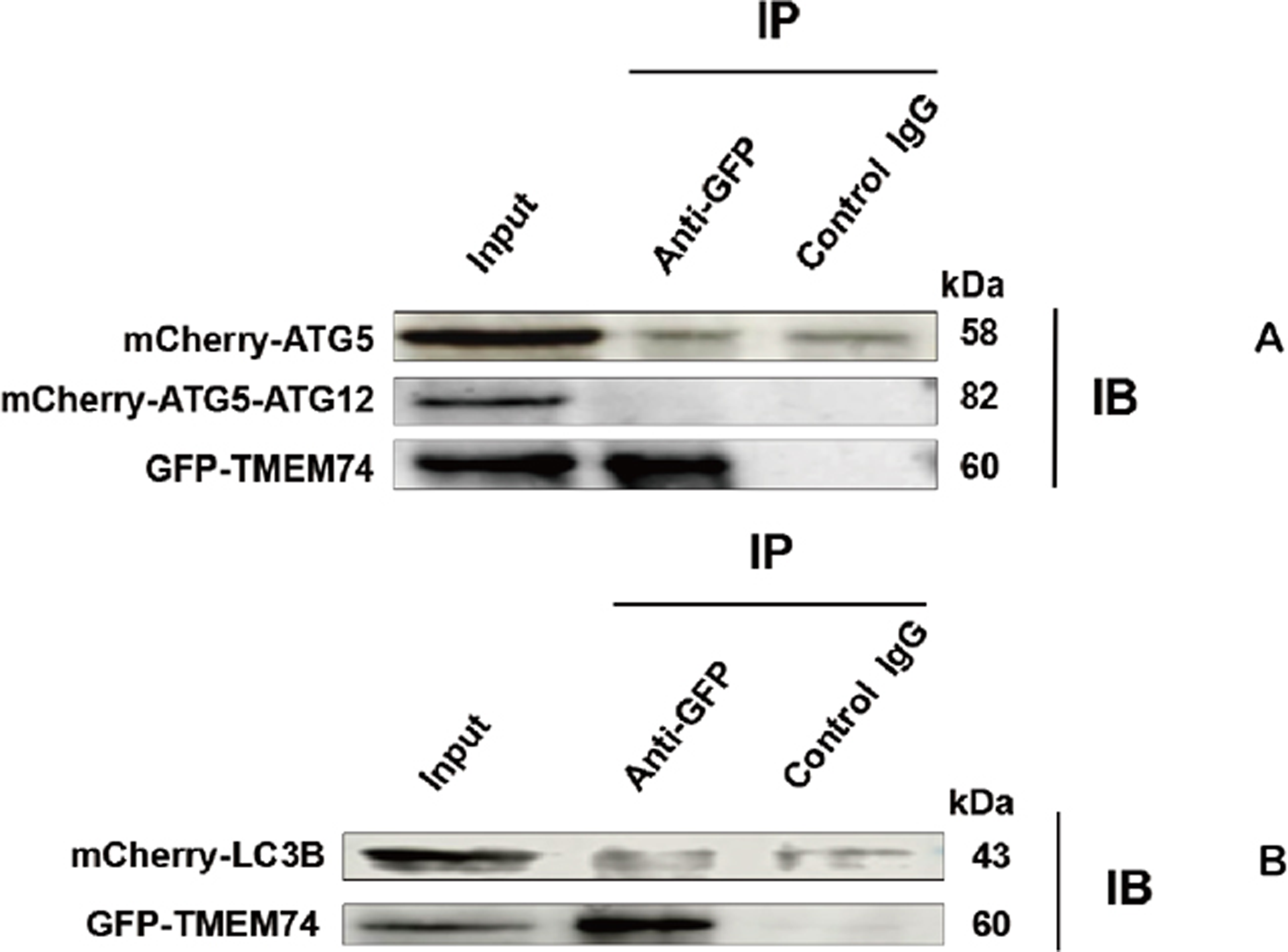

Supplement: Supplementary Figure 4 [file cddis2017370x4.tif]

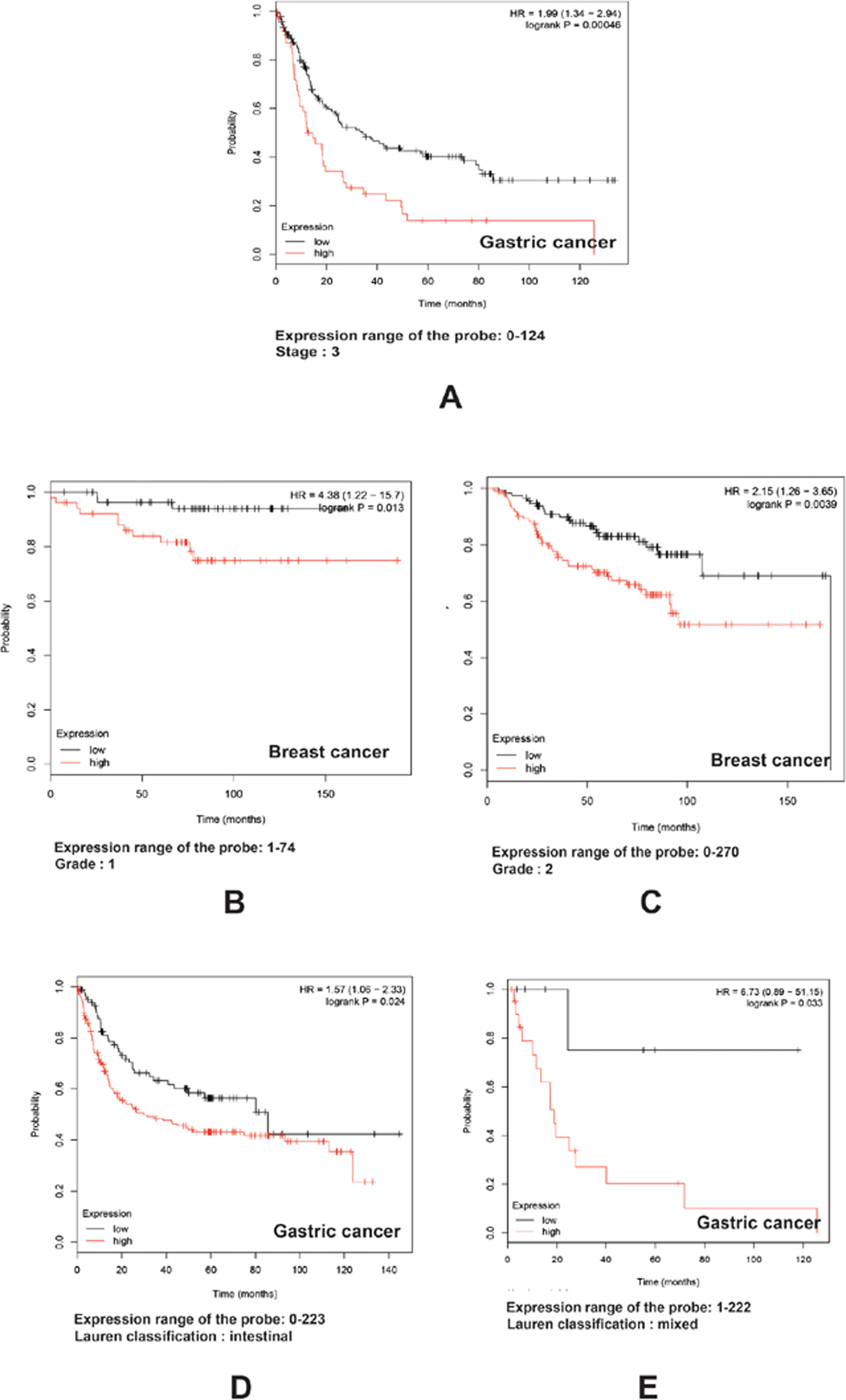

Supplement: Supplementary Figure 8 [file cddis2017370x8.tif]

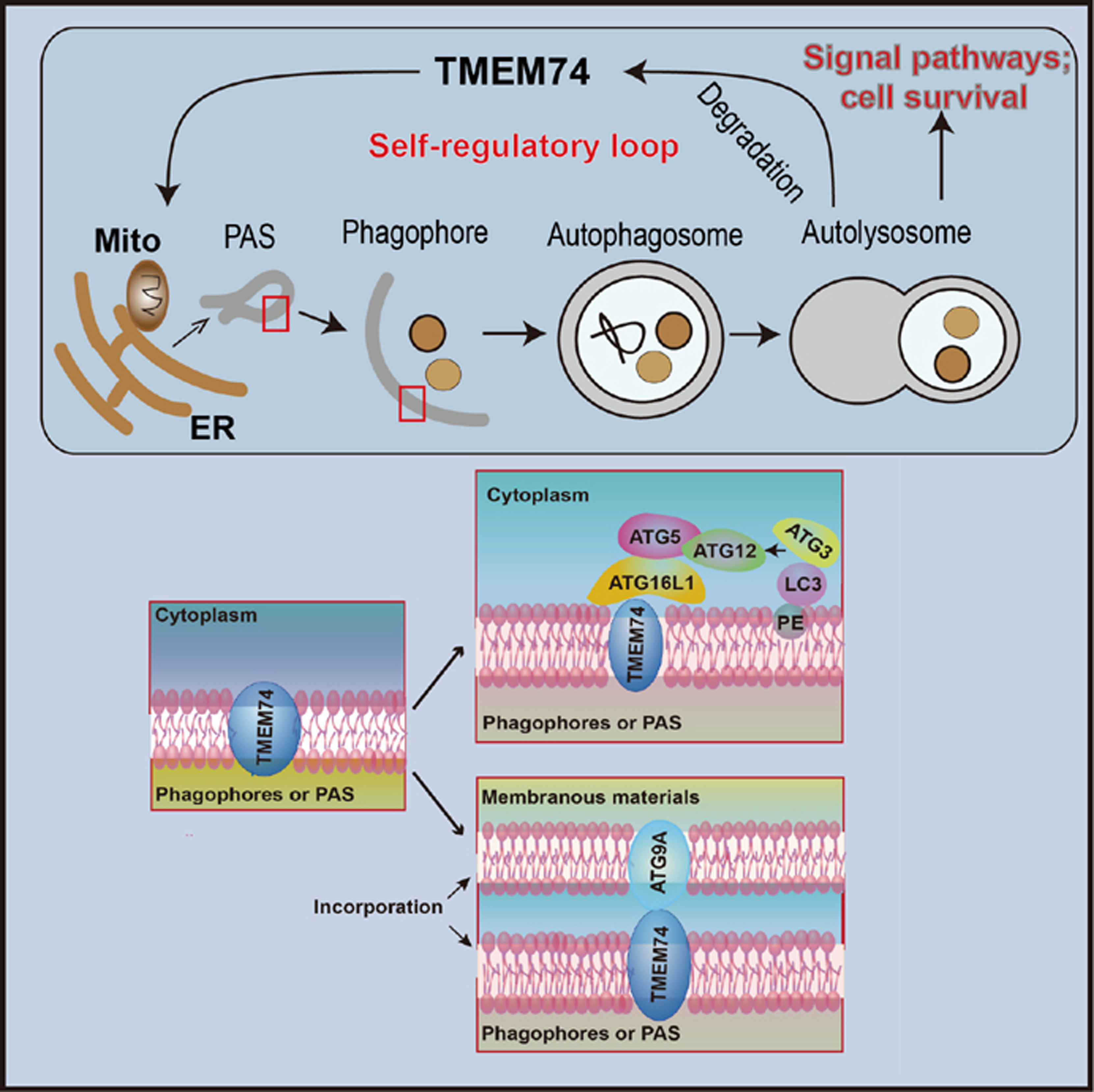

Supplement: Supplementary Figure 9 [file cddis2017370x9.tif]
