# Supplementary material for: TMEM74 promotes tumor cell survival by inducing autophagy via interactions with ATG16L1 and ATG9A
Source: Cell Death Dis. 2017 Aug 31;8(8):e3031–. doi: 10.1038/cddis.2017.370 (PMC5596558; doi:10.1038/cddis.2017.370)
Supplement: Supplementary Figure Legends [file cddis2017370x10.docx]

Supplementary Figure legends

Figure 1S. Knockdown of *TMEM74* blocks the EBSS-induced autophagy in HepG2 and 786-O cells and TM domains of TMEM74 are required for autophagy induction. (A,B) HepG2 cells and 786-O cells were transfected with *siTMEM74-1/siTMEM74-2* or siControl and treated with EBSS for at least 12h.the endogenous LC3B-II levels were detected by western blotting. (C) Truncated mutants, the TMEM74^△TM^ , was constructed. (D) HeLa cells were transfected with GFP(control), GFP-TMEM74.or GFP-TMEM74^△TM^ for 24h, then LC3B-II levels were detected by western blotting. (E,F) HeLa cells were co-transfected with TMEM74 and RFP-LC3B or TMEM74^△TM^ and RFP-LC3B, the RFP-LC3B puncta were observed by confocal microscopy. Quantification of RFP-LC3B punta per cell was shown as column,Data are means±SD of at least 20 cells scored from at least 3 observed regions.**p<*0.05, ***p*<0.01.

Figure 2S. Knockdown of *TMEM74* hampers the LC3 puncta formation and decreases the colocalization between LC3 and ATG16L1 or STX17. (A,B) Representative confocal images were shown in HeLa cells transfected with *SiTMEM74* or SiControl for 24h, subsequently treated with RFP-LC3 and GFP-ATG16L1, the co-localization of RFP-LC3 and GFP-ATG16L1 was observed after 8h EBSS starvation. (C) The quantification of number of RFP-LC3 puncta colocalized with GFP-ATG16L1 in cells treated as in (A and B) . Data are means ± SD of average value per cell from 3 experiments, at least 10 selected regions per experiment. **p<*0.05, ***p*<0.01. (D,E) Representative confocal images were shown in HeLa cells transfected with *SiTMEM74* or SiControl for 24h, subsequently treated with RFP-LC3 and GFP-STX17, the co-localization of RFP-LC3 and GFP-STX17 was observed after 8h EBSS starvation. (F) The quantification of number of RFP-LC3 puncta colocalized with GFP-STX17 in cells treated as in (D and E) . Data are means ± SD of average value per cell from 3 experiments, at least 10 selected regions per experiment. **p<*0.05, ***p*<0.01. (G) The efficiency of knockdown of *TMEM74* was detected by western blotting.

Figure 3S. TMEM74 colocalizes with ER and mitochondria with the time dependence. (A,B) The HeLa cells were co-transfected with GFP-TMEM74 and mCherry-ER or mCherry-Mito respectively for 12h, then observed by the real-time confocal microscopy. The observing zero point was started from 12h after transfection. (C) The result are means±SD of Pearson Correlation of two selected regions. Pearson Correlation reflects the levels of colocalization.

Figure 4S. TMEM74 fails to interact with ATG5-ATG12 and LC3.(A) HeLa cells were co-transfected with GFP-TMEM74 and mCherry-ATG5 for 24h, Total cell extracts were subjected to IP using either an anti-GFP or an isotype control IgG, ATG5 was detected in the washed beads using anti-ATG5 IgG by western blotting.(B), HeLa cells were co-transfected with GFP-TMEM74 and mCherry-LC3B for 24h, Total cell extracts were subjected to IP using either an anti-GFP or an isotype control IgG, LC3B was detected in the washed beads using anti-LC3B IgG by western blotting.

Figure 5S. TMEM74-induced autophagy alters the AKT phosphorylation. (A,B,C,D,E) The levels of AKT phosphorylation were detected from the HeLa cells extracts transfected *SiATG5, SiATG16L1, SiATG7, SiATG3, SiATG10* or SiControl followed by treatment of GFP-TMEM74 or GFP( Control). (F, G) The levels of AKT phosphorylation were detected from the HeLa cells extracts transfected *SiBECN1, SiPI3KC3* or SiControl followed by treatment of GFP-TMEM74 or GFP( Control).

Figure 6S. TMEM74 serves as positive factor for tumor cell survival. (A,B,C,D) HeLa cells were transfected with GFP-TMEM74 or GFP(control) respectively,treated with or without bafilomycin.A1(100nM), glucose-free medium or etoposide (100nM), then monitored the cell proliferation by CCK-8 assay (cell counting kit-8). Data are means±SD of three replicates.**p<*0.05, ***p*<0.01.

Figure 7S. TMEM74 promotes the resistance to metabolic stress in U2OS,HepG2 and 786-O cells (A~F) HepG2, U2OS, and 786-O cells were transfected with Flag-TMEM74 or Flag(control) respectively for 12h, then cultured with glucose-free medium for 12h. the apoptotic cells were measured with AnnexinV-PI dual staining followed by Flow cytometry. Data are means±SD of three experiments.

Figure 8S. The supplementary survival analysis at high or low expression of TMEM74. (A,D,E) The first progression survival periods of stage 3 , intestinal and mixed type gastric cancer were analyzed at different levels of TMEM74.

(B,C) The first progression survival periods of grade 1 and 2 breast cancer were analyzed at different levels of TMEM74.

Figure 9S. The mechanism of TMEM74-induced autophagy and the self-regulatory loop of TMEM74-related autophagy. TMEM74 interacts with ATG16L1 to promote ATG5-ATG12/ATG16L1 complex formation so as to recruit LC3 to PE, which favours the membrane bending(detachment from the source membranes) and expansion (incorporation with other vesicles).

TMEM74 interacts with ATG9A to facilitate the tethering of ATG9A-vesicles to phagophores. Meanwhile, TMEM74 may promote the ATG9A binding to WIPI1 for the retrograde transport.

The common autophagic process is initiated by diverse extra-intra-cellular factors like nutrient deprivation or drug treatment mediated by varied signal pathways. On account of the lack of effective control ways, the common autophagy process easily appears unconstrained unless the negative factors are withdrawn and may intersect with other types of cell death like apoptosis leading to cell death. However, TMEM74 bypasses the upstream signal pathways and molecules and may directly stimulate the nucleation process to induce autophagy, inversely, to affect the upstream signal pathways. Moreover, TMEM74 can be down-regulated by proteasomes and autolysosomes even gene expression regulation, which forms a self-regulatory loop to restrain the autophagy levels, so the antophagic positive effects are displayed completely.
